# Respiratory Acidosis as a Diagnostic Clue in Symptomatic Epilepsy With Stroke Mimics: A Case Report

**DOI:** 10.7759/cureus.62449

**Published:** 2024-06-15

**Authors:** Keita Takahashi, Shigeto Ishikawa, Hiroyuki Takeuchi

**Affiliations:** 1 Department of Emergency Medicine, Fukuoka Kinen Hospital, Fukuoka, JPN

**Keywords:** clinical case report, symptomatic epilepsy, respiratory acidosis, partial epilepsy, stroke mimics

## Abstract

Stroke mimics are difficult to differentiate from each other. Symptomatic epilepsy can also occur, but it is necessary to perform a magnetic resonance imaging (MRI) scan to distinguish it from a stroke. Although respiratory acidosis has been reported to occur with partial-onset seizures due to prolonged apnea, respiratory acidosis is rarely suspected to be a sign of epilepsy. We report a case in which respiratory acidosis helped to diagnose symptomatic epilepsy with stroke mimics. The patient was a 52-year-old female who was brought to the emergency room with the chief complaint of difficulty in talking. When she visited the hospital, sensory aphasia was observed, and a computed tomography (CT) scan was performed. She vomited after the CT scan, and an arterial blood gas analysis showed a pH of 7.26 with a PaCO_2_ level of 71 mmHg, indicating respiratory acidosis. After the administration of diazepam, the seizures abated and her sensory aphasia improved. Later, an investigation of the patient’s history revealed symptomatic epilepsy and discontinuation of antiepileptic drugs. If unexplained respiratory acidosis is noted in a patient with stroke mimics, a further investigation of the patient’s history and physical examination may help to diagnose symptomatic epilepsy.

## Introduction

Epileptic seizure patterns can manifest as various symptoms [1]. Stroke mimics can be difficult to distinguish from stroke; therefore, it is important to confirm a patient's medical history and perform imaging tests [2]. In the case of cerebral infarction, there is a tendency to rush the imaging examination due to the time limit for starting thrombolytic therapy; however, it is important to ensure that the patient is stable.

Epileptic seizures are known to cause metabolic acidosis due to lactic acid accumulation caused by excessive muscle activity, high body temperature, and anaerobic metabolism [1]. Respiratory acidosis rarely raises the suspicion of epilepsy. In the present case, a patient with a history of symptomatic epilepsy after cerebral hemorrhage was brought to the emergency department with stroke mimics and was found to have respiratory acidosis. The patient subsequently developed seizures, which led to a diagnosis of symptomatic epilepsy. We report the relationship between respiratory acidosis and symptomatic epilepsy, with a discussion of the relationship between respiratory acidosis and epilepsy.

## Case presentation

The patient was a 52-year-old woman. Her family noticed that she was not engaging in telephone conversations and called for emergency medical assistance. On arrival at the hospital, she had sensory aphasia, which had been present for two hours since the onset of aphasia. Her vital signs were as follows: Glasgow Coma Scale, E4V3M6; heart rate, 122 beats/min; blood pressure, 148/89 mmHg; SpO_2_, 95% (room air); respiratory rate, 10 breaths/min; and body temperature, 36.6°C. On physical examination, her pupils were 4/4 mm in size and there was a light reflex. There was no co-divergence or drooping at the corners of the mouth. No obvious motor paralysis or neck rigidity was observed. An arterial blood gas analysis showed respiratory acidosis with a pH of 7.32 and a PaCO_2_ level of 62 mmHg (Table 1).

**Table 1:** Results of arterial blood gas analyses (at the time of admission)

Parameter	Result	Reference Range
pH	7.32	7.35-7.45
PaCO_2_	62	35-45 (mmHg)
PaO_2_	62	80-100 (mmHg)
Lactate	1.1	0.5-1.6 (mmol/L)
Bicarbonate ion	31.9	22-26 (mmol/L)
Base excess	4.1	-2.0 to 2.0 (mmol/L)
Glucose	172	73-109 (mg/dL)

Laboratory test results revealed no significant abnormalities (Table 2).

**Table 2:** Laboratory data WBC, white blood cells; Hb, hemoglobin; Plt, platelets; Alb, albumin; T-Bil, total bilirubin; AST, aspartate aminotransferase; ALT, alanine aminotransferase; BUN, blood urea nitrogen; Cr, creatinine; Glu, glucose; CRP, C-reactive protein; Na, sodium; K, potassium; Cl, chloride.

Parameter	Result	Reference value
WBC	5010	3300-8600 (/µL)
Hb	13.2	11.6-14.8 (g/dL)
Plt	23.1	15.8-34.8 (×10^4^/µL)
Alb	4.1	4.1-5.1 (g/dL)
T-Bil	0.43	0.4-1.5 (mg/dL)
AST	27	13-30 (U/L)
ALT	19	7-23 (U/L)
BUN	11	8.0-20.0 (mg/dL)
Cr	0.68	0.46-0.79 (mg/dL)
Na	147	138-145 (mEq/L)
K	4.3	3.6-4.9 (mEq/L)
Cl	110	101-108 (mEq/L)
Glu	153	73-109 (mg/dL)
CRP	0.02	0.00-0.14 (mg/dL)

**Figure 1:**
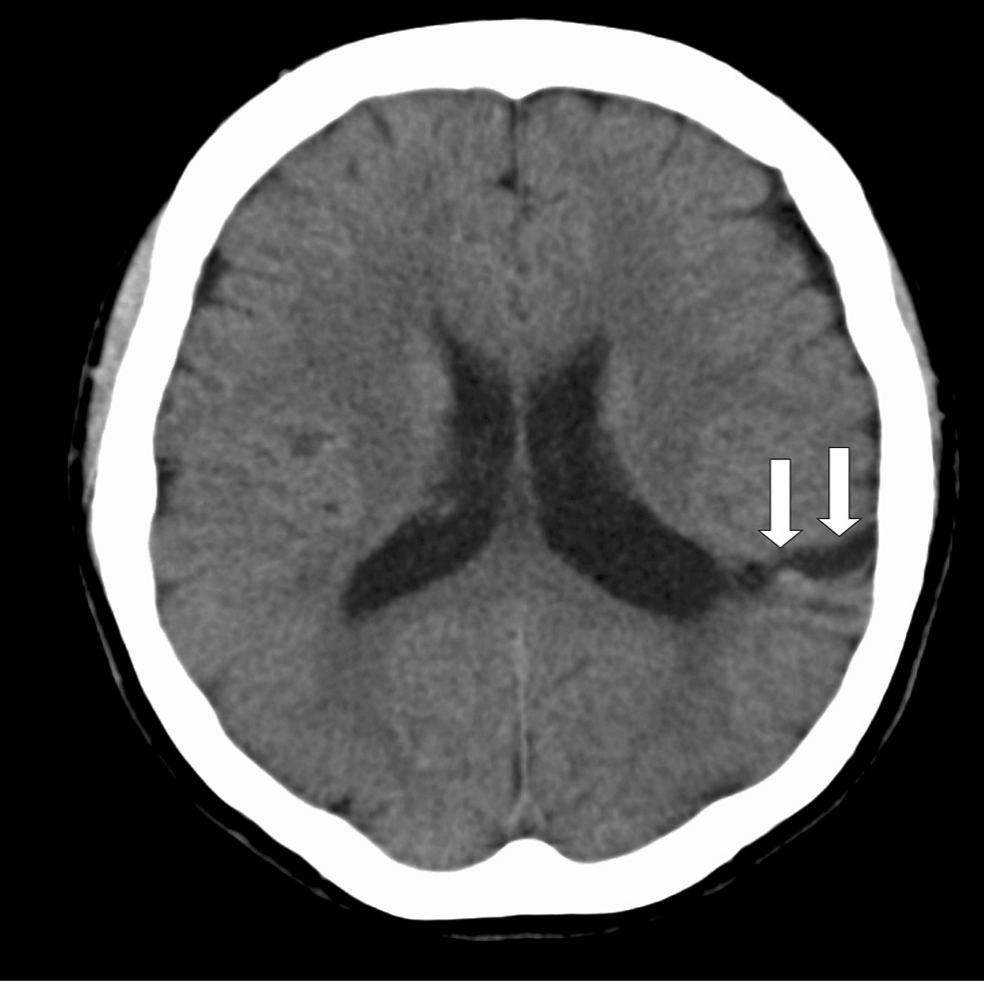
Head CT image Head computed tomography revealed a hypo-absorptive zone in the left temporal lobe (shown by white arrows), which is considered to be a finding of cerebral hemorrhage.

Chest radiography through a computed tomographic (CT) scan revealed no cardiac enlargement or abnormalities in the lung fields (Figure 1).

The patient vomited and her SpO_2_ level dropped to 88%. Therefore, oxygen was administered at 2 L/min. An arterial gas analysis showed a pH of 7.26, a PaCO_2_ level of 71 mmHg, and progressive respiratory acidosis (Table 3).

**Table 3:** Results of arterial blood gas analyses (after CT imaging (nasal oxygen 2 L/min))

Parameter	Result	Reference Range
pH	7.26	7.35-7.45
PaCO_2_	71	35-45 (mmHg)
PaO_2_	165	80-100 (mmHg)
Lactate	0.8	0.5-1.6 (mmol/L)
Bicarbonate ion	31.9	22-26 (mmol/L)
Base excess	2.9	-2.0-2.0 (mmol/L)
Glucose	150	73-109 (mg/dL)

Considering that the vomiting occurred due to cerebral infarction, metoclopramide (10 mg) was administered. During a magnetic resonance imaging (MRI) examination, the patient vomited again and generalized convulsions were observed. The MRI scan was canceled. After the administration of diazepam (10 mg), the convulsions ceased and the patient's sensory aphasia improved.

Subsequent medical history revealed that levetiracetam (500 mg), which had been prescribed for symptomatic epilepsy following cerebral hemorrhage, had been discontinued four months earlier. The seizures were diagnosed as having shifted from partial seizures to generalized seizures due to symptomatic epilepsy, and the plan was to administer lacosamide (100 mg) and restart levetiracetam after hospitalization. The patient was discharged on the third day of hospitalization without any further epileptic seizures.

## Discussion

Neuropathic diseases and hypoglycemia are the differential diagnoses of stroke mimics. Symptomatic epilepsy is reported to account for 20.4% of these cases [3]. MRI is a key diagnostic tool for differentiating cerebral infarction from stroke [2]. It is important that the diagnosis of cerebral infarction by MRI and the time of onset within 4.5 hours be inferred from the mismatch between diffusion-weighted imaging (DWI) and fluid-attenuated inversion recovery (FRAIR) mismatch as criteria for the indication of alteplase in thrombolytic therapy [4]. The patient must rest during the MRI examination and must be in a stable condition for the examination to be performed. In the present case, the patient presented to our hospital within two hours of the onset of symptoms, and we decided to proceed with the MRI examination early to investigate stroke. Vomiting can be a symptom of symptomatic epilepsy.

Metabolic acidosis due to lactic acid accumulation is known to occur during epileptic seizures [1], and it has been pointed out that apnea occurs in temporal lobe epilepsy due to the spillover of seizures to the contralateral hemisphere [5]. Respiratory acidosis has also been reported to be caused, in part, by increased intrapulmonary shunting and transient neurogenic pulmonary edema due to epileptic seizures [6]. In addition, hypoxemia and respiratory acidosis are known to induce symptomatic epilepsy [7]. Therefore, when respiratory acidosis is detected, the possibility of partial seizures leading to generalized seizure should be considered. Although this is an important finding, the most common differential diagnoses to consider when examining patients with respiratory acidosis in the emergency department are acute respiratory distress syndrome, chronic obstructive pulmonary disease, asthma attacks, and acute heart failure; epileptic seizures are not common [8]. In addition, the author found only one case of stroke mimicking respiratory acidosis in a PubMed search for "stroke mimics" and "respiratory acidosis" [9].

In the present case, a physical examination and chest radiography showed no signs of any diseases causing respiratory failure. Although we rushed the MRI scan to rule out stroke, a search for the cause should be performed when respiratory acidosis is present and when a patient with a history of symptomatic epilepsy presents with stroke mimics. The patient’s history should have been taken appropriately, and antiepileptic drugs could have been administered to rule out the possibility of symptomatic epileptic seizures or partial-onset seizures, thereby allowing for a safe examination and avoiding unnecessary MRI testing.

## Conclusions

Partial seizures can cause stroke mimics, which are difficult to diagnose. In the present case, the patient’s history was retaken and another physical examination was conducted when the patient was found to have respiratory acidosis, which led to the diagnosis of epilepsy and allowed imaging studies to be safely performed.
